# Cooperative and Competitive Reinforcement and Imitation Learning for a Mixture of Heterogeneous Learning Modules

**DOI:** 10.3389/fnbot.2018.00061

**Published:** 2018-09-27

**Authors:** Eiji Uchibe

**Affiliations:** Advanced Telecommunications Research Computational Neuroscience Laboratories, Department of Brain Robot Interface, Kyoto, Japan

**Keywords:** reinforcement learning, imitation learning, modular architecture, parallel learning, entropy-regularization, multiple importance sampling

## Abstract

This paper proposes Cooperative and competitive Reinforcement And Imitation Learning (CRAIL) for selecting an appropriate policy from a set of multiple heterogeneous modules and training all of them in parallel. Each learning module has its own network architecture and improves the policy based on an off-policy reinforcement learning algorithm and behavior cloning from samples collected by a behavior policy that is constructed by a combination of all the policies. Since the mixing weights are determined by the performance of the module, a better policy is automatically selected based on the learning progress. Experimental results on a benchmark control task show that CRAIL successfully achieves fast learning by allowing modules with complicated network structures to exploit task-relevant samples for training.

## 1. Introduction

Reinforcement Learning (RL) (Sutton and Barto, 1998; Kober et al., 2013) is an attractive learning framework with a wide range of possible application areas. A learning agent attempts to find a policy that maximizes its total amount of reward received during interaction with its environment. Recently, such nonlinear function approximators as artificial neural networks are being used to approximate a policy with the help of deep learning. Deep Reinforcement Learning (DRL), which integrates both deep learning and reinforcement learning, has achieved several remarkable successes in decision-making tasks, such as playing video games (Mnih et al., 2015) and the board game Go (Silver et al., 2016, 2017).

However, DRL's performance critically depends on its architectures, learning algorithms, and meta-parameters (Henderson et al., 2018). On one hand, a shallow Neural Network (NN) with fewer connection weights usually learns faster, but its performance may be limited. A deep and/or wide NN with many network weights can represent any complex policy, but it usually needs a huge amount of experiences to find an appropriate one. Since the motivation to use NNs is to represent complicated nonlinear mapping from state to action, it is reasonable to select a deep and wide NN as a function approximator. However, training data must be gathered by the learning agent for reinforcement learning as opposed to the standard settings of the classification problems of deep learning. Since a complicated NN policy whose many weights are initialized randomly does not collect useful experiences to seek its goal, it is not promising to collect good experiences by itself, especially at the beginning of the learning. Therefore, we have to find an appropriate network architecture based on the task's complexity. Although an evolutionary method was applied to the problem of a neural architecture search (Whiteson and Stone, 2006) for tiny problems, experimenters usually manually prepare a learning module with an appropriate network architecture depending on the situation. Furthermore, it is crucial to select an appropriate RL algorithm based on the given task. For instance, two major types of algorithms exist: value-based reinforcement learning and policy search methods, including policy gradient reinforcement learning. Such value-based reinforcement learning as Q-learning (Watkins and Dayan, 1992) and SARSA (Rummery and Niranjan, 1994) learns faster than vanilla policy search methods such as REINFORCE (Williams, 1992) because value-based reinforcement learning exploits the Bellman equation under the Markovian assumption. The policy search methods are robust and find a better stochastic policy even if the state representation is deficient (Kalyanakrishnan and Stone, 2011).

In practice, experimenters test different combinations to select the best one since their appropriate combination is unknown in advance. Moreover, since the sequential testing of these factors is very time-consuming, to eliminate the need for such human hand-tuning, we proposed Cooperative and competitive Learning with Importance Sampling (CLIS) (Uchibe and Doya, 2004, 2005). Here, the agent possesses multiple heterogeneous learning modules and selects an appropriate module based on the task and its experience. We consider a mechanism by which an agent can best utilize its behavioral experiences to train multiple learning modules with different network architecture and learning algorithms. By exploiting task-relevant experiences gathered by suboptimal but fast-learning modules, a complicated module learns faster than when it was trained alone. Unfortunately, CLIS is unstable in learning for several reasons. One is the naive use of importance sampling to compensate for the mismatch in the target and behavior policies. The other is that the original CLIS adopts classical RL algorithms and linear function approximators. In addition, the application of CLIS to robot control is quite limited because it is implicitly assumed that the action is discrete.

To overcome the problems raised by the study of CLIS, this paper proposes Cooperative and competitive Reinforcement And Imitation Learning (CRAIL), which extends CLIS to stabilize learning processes and improve sampling efficiency. Similar to CLIS, CRAIL maintains a set of multiple heterogeneous policies, including hand-coded controllers, and collects samples by a behavior policy constructed by the mixture distribution of the policies. Because the mixing weights are computed by the performance of the module, a better policy is automatically selected based on the learning progress. Then all the modules are trained simultaneously by two objective functions. CRAIL introduces the following two components to CLIS: (1) multiple importance sampling, and (2) policy learning using a combination of temporal difference and behavior cloning loss. Using multiple importance sampling stabilizes the learning process of the policy search methods because the correction factor, which is called the importance-sampling ratio, is upper-bounded. One critical contribution of CRAIL is its introduction of behavior cloning loss as well as temporal difference learning. Based on the learning processes of multiple modules, CRAIL dynamically updates the behavior policy that will be used as the best expert policy. Unlike learning from demonstrations, we can explicitly compute the behavior cloning loss based on a behavior policy, which significantly improves the policy updates. Furthermore, we use modern reinforcement learning algorithms such as entropy-regularized RL because of several advantages described later.

We compare CRAIL with CLIS on four benchmark control tasks supported by the OpenAI gym (Brockman et al., 2016). Experimental results indicate that by exploiting task-relevant episodes generated by suboptimal, but fast-learning modules a complex learning module trained with CRAIL actually learns faster than when it is trained alone. Due to adding the behavior cloning loss, CRAIL learns much faster than CLIS on all the benchmark tasks. In addition, CRAIL effectively transfers samples collected by the fixed hand-coded controller to train policies implemented by neural networks.

## 2. Related work

Several reinforcement learning methods with multiple modules have been proposed. Compositional Q-learning (Singh, 1992) selects a learning module with the least TD-error, and Selected Expert Reinforcement Learner (Ring and Schaul, 2011) extends the value function to select a module with better performance. Doya et al. (2002) proposed Multiple Model-based Reinforcement Learning (MMRL), in which each module is comprised of a state prediction model and the module with the least prediction error is selected and trained. These approaches are interpreted as the concept of “Mixture of Experts.” In these approaches, the structure of each module is the same and uses the same learning algorithm, while CRAIL enables the use of heterogeneous learning modules that can be trained concurrently. One interpretation is that the modules are spatially distributed in their methods because they change the module based on the current environmental state. On the other hand, CRAIL temporarily distributes the modules because it switches them due to the learning progress.

Some researchers integrated an RL algorithm with hand-coded policies to improve the learning progress in its initial stage. Smart and Kaelbling (2002) proposed an architecture comprised of a supplied control policy and Q-learning. In the first learning phase, a robot was controlled with the supplied control policy developed by a designer. The second learning phase begins to control the robot effectively when the value function is approximated sufficiently. Xie et al. (2018) proposed a similar approach to incorporate *a prior* knowledge, in which Deep Deterministic Policy Gradient (DDPG) (Lillicrap et al., 2016) and a PID controller are used as off-policy learning and a hand-coded policy, respectively. However, a limitation of their approach is that it uses only one learning module. CRAIL is a more general architecture for incorporating multiple prior knowledge. In addition, it can automatically select an appropriate module depending on the learning progress. Sutton et al. (1999) described the advantages of off-policy learning and proposed a novel framework to accelerate learning by representing policies at multiple levels of temporal abstraction. Although their method assumed a semi-Markov decision problem and AVRL, CLIS can use different learning algorithms.

Our framework can be interpreted as learning from demonstrations. Many previous studies can be found in this field, and some recent studies such as (Gao et al., 2018; Hester et al., 2018; Nair et al., 2018) integrated reinforcement learning with learning from demonstrations by augmenting the objective function. Our framework resembles those methods from the viewpoint of the design of the objective function. The role of the demonstrator is different because our framework's demonstrator is selected from multiple heterogeneous policies based on the learning progress; previous studies assumed that it is stationary and used it to generate a training dataset. Since CRAIL explicitly represents the behavior policy, actions can be easily sampled from it to evaluate the behavior cloning loss.

The most closely related study is Mix & Match (Czarnecki et al., 2018), in which multiple heterogeneous modules are trained in parallel. Mix & Match's basic idea resembles CRAIL, but it does not consider multiple reinforcement learning algorithms; CRAIL adopts three learning algorithms for every module. In addition, Mix & Match uses a mixture of policies and optimizes the mixing weights by a kind of evolutionary computation. Since Mix & Match needs multiple simulators, it is sample-inefficient. The mixing weights are automatically determined in the case of CRAIL.

## 3. Methods

### 3.1. CRAIL's architecture

**Figure 1:**
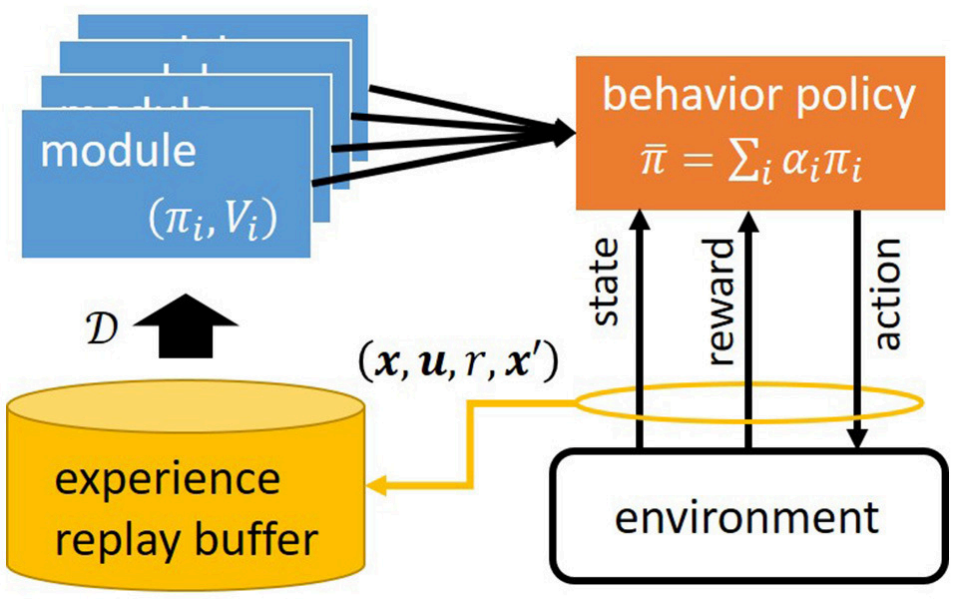
Architecture of Cooperative and Competitive Reinforcement And Imitation Learning (CRAIL).

We investigate the standard Markov Decision Process (MDP) framework, which is not known by an agent in the model-free RL setting (Sutton and Barto, 1998). An MDP is formulated as follows: (1) X is the state space and ${\text{x}}_{t}\in X$ denotes the state of the environment at time *t*; (2) U is the action space and $u_{t}\in U$ is the action executed by the agent at time *t*; (3) $p_{e}\left(x^{\prime }|x,u\right)$ is the state transition probability for $x,x^{\prime }\in X$ and u∈U; (4) *p*_0_(***x***) is the initial state probability; and (5) *r*(***x***, ***u***) is a reward function. CRAIL has *M* learning modules as shown in Figure 1, and each of which has state value function *V*_*i*_(***x***; **ψ**_*i*_), state-action value function *Q*_*i*_(***x***, ***u***; **θ**_*i*_), and policy π_*i*_(***u***∣***x***; **ϕ**_*i*_), where **ψ**_*i*_, **θ**_*i*_, and **ϕ**_*i*_ are the parameters, respectively. *V*_*i*_ and *Q*_*i*_ are defined as a discounted sum of the rewards given by

$$\begin{array}{l} \;V_{i}(x)=E\left[\sum_{t=0}^{\infty }\gamma^{t}r(x_{t},u_{t})|x_{0}=x\right],\\ Q_{i}(x,u)=E\left[\sum_{t=0}^{\infty }\gamma^{t}r(x_{t},u_{t})|x_{0}=x,u_{0}=u\right], \end{array}$$

where γ ∈ [0, 1) is a discount factor that determines the relative weighting of immediate versus later rewards. For simplicity, all the modules share the same sensory-motor system.

At each time step *t*, the agent selects an action based on the following behavior policy:

(1) $$\bar{\pi }(u_{t}|x_{t})=\sum_{i=1}^{M}\alpha (i|x_{t})\pi_{i}(u_{t}|x_{t};\phi_{i}).$$

Because the state value function evaluates the policy's performance, we use it to determine the mixing weight:

(2) $$\alpha (i|x_{t})=\frac{\exp(\beta V_{i}(x_{t};\psi_{i}))}{\sum_{j=1}^{M}\exp(\beta V_{i}(x_{t},\psi_{j}))},$$

where β is an inverse temperature. A low β value causes (most of) the equiprobable selection of all the modules, while its high value causes the selection of a module with the highest value when the probability comes closest to one. Inverse temperature β plays an important role at the early stage of learning concerning whether to select optimistic modules that may have large initial values. Algorithm **1** illustrates an overview of the learning process of stepwise CRAIL. The agent maintains experience replay buffer D to store state transition (***x***, ***u***, ***r***, ***x***′) by behavior policy $\bar{\pi }$.

**Algorithm 1:** Stepwise CRAIL

1: Initialize all parameters of the learning modules.
2: Initialize empty replay buffer D.
3: **repeat**
4: ***x***_0_~*p*_0_(·) ⊳ Draw an initial state.
5: **for** *t* = 0, …, *T*−1 **do**
6: ${\text{u}}_{t}\sim \bar{\pi }\left(\cdot |{\text{x}}_{t}\right)$, ***x***_*t*+1_, *r*_*t*_~*p*_*e*_(·, ·∣***x***_*t*_, ***u***_*t*_).
7: Add batch data {***x***_*t*_, ***u***_*t*_, *r*_*t*_, ***x***_*t*+1_} to replay buffer D.
8: **for** *i* = 1, …, *M* **do** ⊳ Update all the modules.
9: update the parameters by Algorithms 4 or **5**.
10: **end for**
11: **end for**
12: **until** convergence

As a special case for episodic tasks, we focus on episodic CRAIL, which is basically identical to the original CLIS, as shown in Algorithm **2**. At the beginning of every episode, a module is chosen by Equation (2) to generate a sequence of states, actions, and rewards denoted by

$$h≜[x_{1},u_{1},r_{1},\ldots ,x_{T},u_{T},r_{T}],$$

where *T* denotes the number of steps called the horizon length. This modification is useful from the viewpoint of numerical stability when a hand-coded deterministic policy is used as domain knowledge. For example, a Central Pattern Generator (CPG) is widely used to generate rhythmic motions like walking without rhythmic sensory inputs (Ijspeert, 2008), but it cannot be represented by policy π_*i*_(***u***∣***x***) because CPG has internal states that are not observable by other modules. In this case, the module has to cope with partially observable MDP tasks if the experiences generated by the CPG-based controller are used for training.

**Algorithm 2:** Episodic CRAIL/CLIS

1: Initialize all parameters of the learning modules.
2: Initialize empty replay buffer D.
3: **repeat**
4: **for** *k* = 1, …, *K* **do** ⊳ Collect *K* episodes
5: ***x***_0_~*p*_0_(·) ⊳ Draw an initial state.
6: *i*~α(·∣***x***_0_) ⊳ Select a module.
7: **for** *t* = 0, …, *T*−1 **do**
8: ***u***_*t*_~π_*i*_(·∣***x***_*t*_), ***x***_*t*+1_, *r*_*t*_~*p*_*e*_(·, ·∣***x***_*t*_, ***u***_*t*_).
9: **end for**
10: Add batch data {*i*, ***x***_0:*T*_, ***u***_0:*T*−1_, *r*_0:*T*−1_} to replay buffer D.
11: **for** *i* = 1, …, *M* **do** ⊳ Update all the modules.
12: update the parameters by Algorithms 3, **4**, or **5**.
13: **end for**
14: **end for**
15: **until** convergence

### 3.2. Learning algorithm in each module

Similar to CLIS, all the modules learn an optimal policy in parallel on the samples from D collected by the behavior policy. The learning algorithms used by CRAIL should be able to learn from the experiences gathered by other modules, and therefore, we adopt the following three methods as an off-policy RL algorithm: REINFORCE (Williams, 1992), Soft Actor-Critic (Soft AC) (Haarnoja et al., 2018), and Deterministic Policy Gradient (DPG) (Lillicrap et al., 2016). We modify these algorithms by incorporating behavior loss to update the policy to improve their learning efficiency.

#### 3.2.1. REINFORCE with importance sampling

Policy search methods that do not rely on the Bellman optimality equation such as REINFORCE (Williams, 1992) have been reevaluated because of their simplicity and robust performance with non-Markovian tasks (Meuleau et al., 1999). REINFORCE is essentially an on-policy method (Sutton and Barto, 1998) because it estimates the gradient at a particular point in the policy space by acting precisely in the manner of its corresponding policy during learning trials. To use samples collected by the behavior policy, we introduce importance sampling to the REINFORCE algorithm (Meuleau et al., 2001) as an off-policy learning algorithm. Note that REINFORCE is applicable for the episodic CRAIL because it requires a set of sequences as a dataset.

REINFORCE evaluates sequence *h* by

$$J_{i}^{\pi }(\phi_{i},h)=R(h)=\sum_{t=1}^{T}\gamma^{t-1}r_{t},$$

where *R*(*h*) is called the return, which is defined as the discounted sum of rewards along *h*. To update **ϕ**_*i*_, REINFORCE adopts the stochastic gradient ascent method with the gradient given by

(3) $$\frac{\partial J_{i}^{\pi }(\phi_{i},h)}{\partial \phi_{i}}=(R(h)-b)\rho_{i}(h)\sum_{t=1}^{T}\frac{\partial \ln\pi_{i}(u_{t}|x_{t})}{\partial \phi_{i}},$$

where *b* is a baseline parameter for variance reduction and ρ_*i*_(*h*) is the importance-sampling weight ratio to account for the change in the distribution, defined by

(4) $$\rho_{i}(h)=\prod_{t=1}^{T}\rho_{i}(x_{t},u_{t})=\prod_{t=1}^{T}\frac{\pi_{i}(u_{t}|x_{t})}{\bar{\pi }(u_{t}|x_{t})},$$

under the Markovian assumption. Unlike CLIS, CRAIL uses multiple importance sampling in which the denominator in (4) is the mixture distribution (1) and therefore ρ_*i*_ is upper-bounded. Note that Equation (3) is slightly different from the standard expression because the expected value with respect to all possible sequences should be considered to exploit the baseline and importance sampling. We will take expectations later to clarify how the gradient of our method is different from the original one.

Although the gradient estimator (3) is sample-efficient, it is close to zero when π_*i*_ is far from $\bar{\pi }$. This situation is often observed at the early stage of learning. To overcome this problem, we introduce the following additional objective function given by the KL divergence between the learning and behavior policies:

(5) $$J_{i}^{\text{BC}}(\phi_{i},x_{t})=D_{\text{KL}}(\bar{\pi }(\cdot |x_{t})∥\pi_{i}(\cdot |x_{t})).$$

Minimizing (5) is behavior cloning, which is also known as supervised imitation learning. However, our method is more computationally efficient because we can draw samples from $\bar{\pi }$ without interacting through the environment. Consequently, the gradient to train the policy parameter is given by

(6) $$\frac{\partial J_{i}^{\pi }(\phi_{i})}{\partial \phi_{i}}=E_{h\sim D}\left[\frac{\partial J_{i}^{\pi ,\text{RL}}(\phi_{i},\cdot )}{\partial \phi_{i}}\right]-\eta E_{x\sim D}\left[\frac{\partial J_{i}^{\pi ,\text{BC}}(\phi_{i},\cdot )}{\partial \phi_{i}}\right],$$

where η is a positive meta-parameter. When η = 0, Equation (6) is identical to the original gradient estimator of REINFORCE with importance sampling.

Finally, state value function *V*_*i*_(***x***, **ψ**_*i*_) is also trained with the Monte Carlo method because it is used to construct the behavior policy. When the number of sequences in D is denoted by *K*, the loss function to optimize the state value function is given by

(7) $$J_{i}^{V}(\psi_{i})=\frac{1}{2}\sum_{k=1}^{K}\sum_{t=1}^{T}{\left(V_{i}(x_{t}^{k})-Y_{t}^{k}\right)}^{2},$$

where $Y_{t}^{k}$ is the target value defined as

$$Y_{t}^{k}=\prod_{t^{\prime }=t}^{T}\rho (x_{t}^{k},u_{t}^{k})\sum_{t^{\prime }=t}^{T}\gamma^{t^{\prime }-t}r_{t}^{k}.$$

The update rule of the modified REINFORCE with importance sampling is given in Algorithm (**3**).

**Algorithm 3:** REINFORCE with importance sampling and Imitation Learning

**Require:** dataset D
1: Sample a random minibatch of sequences *h* from D.
2: Evaluate gradient $\partial J_{i}^{\pi ,\text{RL}}/\partial \phi_{i}$.
3: Sample a random minibatch of states ***x*** from D and ***u*** from $\bar{\pi }$, respectively.
4: Evaluate gradient $\partial J_{i}^{\pi ,\text{BC}}/\partial \phi_{i}$.
5: Update **ϕ**_*i*_ by the stochastic gradient ascent method with Equation (6).
6: Update **ψ**_*i*_ by minimizing Equation (7).

#### 3.2.2. Soft actor-critic and imitation learning

The original CLIS adopted SARSA (Rummery and Niranjan, 1994) with importance sampling (Precup et al., 2001) as an off-policy value-based reinforcement learning algorithm. An advantage is that the technique called eligibility traces (Sutton and Barto, 1998) can be used to accelerate the speed of learning, and it was experimentally shown that deep SARSA can achieve a comparable performance to DQN even though it does not exploit the method of experience replay and target network (Elfwing et al., 2018). However, SARSA implicitly assumes that action is discrete because the stochastic policy must be derived from the state-action value function. Since we are interested in robot control, action must be continuous. Therefore, we adopt Soft Actor-Critic (Haarnoja et al., 2018) as an off-policy algorithm using the value function. Soft Actor-Critic augments the reward function to replace the max-operator with a differentiable one. The reward function is assumed to be given by the following form:

(8) $$\tilde{r}(x,u)=r(x,u)+\frac{1}{\alpha }ℋ(\pi_{i}(\cdot |x)),$$

where α is a positive meta-parameter and and H(π(·∣x)) is the (differential) entropy of policy π_*i*_. Assuming reward function (8), an optimal state value function satisfies the following Bellman optimality equation:

(9) $$V_{i}(x)=\max_{\pi_{i}}E_{\pi_{i}}\left[r(x,u)-\frac{1}{\alpha }\ln\pi_{i}(u|x)+\gamma E_{P_{T}}\left[V_{i}(x^{\prime })\right]\right].$$

The right hand side of Equation (9) is a constrained optimization problem given by

$$\underset{\pi_{i}}{\max}\int \text{d}u\pi_{i}(u|x)\left[r(x,u)-\frac{1}{\alpha }\ln\pi_{i}(u|x)+\gamma E_{P_{T}}\left[V_{i}(x^{\prime })\right]\right],$$

subject to ∫ d***u***π_*i*_(***u*** ∣ ***x***) = 1. In this case, we can analytically maximize the right hand side of Equation (9) by a method with Lagrange multipliers. Consequently, the optimal state value function can be represented by

(10) $$V_{i}(x)=\frac{1}{\alpha }\ln\int \text{d}u\left[\exp(\alpha Q_{i}(x,u))\right],$$

and the corresponding optimal policy can be derived:

(11) $$\pi_{i}(u|x)=\frac{\exp\left(\alpha Q_{i}(x,u)\right)}{\exp(\alpha V_{i}(x))},$$

where state-action value function *Q*(***x***, ***u***) is defined by

(12) $$Q_{i}(x,u)=r(x,u)+\gamma E_{P_{T}}\left[V_{i}(x^{\prime })\right].$$

Note that the right hand side of Equation (10) uses the log-sum-exp operator if the action is discrete, and it is characterized as the “soft” max operator.

The learning algorithm of the Soft Actor-Critic is derived from Equations (10)–(12). Since Equation (12) corresponds to the Bellman optimality equation regarding the state-action value function, it can be used to train parameter **θ**_*i*_ by minimizing the soft Bellman residual for all possible (***x***, ***u***,***x***′) in buffer D:

$$J_{i}^{Q}(\theta_{i},x,u,r,x^{\prime })=\frac{1}{2}{\left\{Q_{i}(x,u)-\left(r+\gamma V_{i}(x^{\prime };{\bar{\psi }}_{i})\right)\right\}}^{2},$$

where $V_{i}\left(\text{x},{\bar{\psi }}_{i}\right)$ and ${\bar{\psi }}_{i}$ respectively denote the target state value network and an exponentially moving average of the parameter vector, which stabilizes the learning used in DQN (Mnih et al., 2015). Consequently, the loss function for training **θ**_*i*_ is given by

(13) $$J_{i}^{Q}(\theta_{i})=E_{(x,u,r,x^{\prime })\sim D}\left[J_{i}^{Q}(\theta_{i},\cdot ,\cdot ,\cdot ,\cdot )\right],$$

where $\left(\text{x},\text{u},r,{\text{x}}^{\prime }\right)\sim D$ means that the transition data are drawn from D.

When the action is discrete, the optimal policy and the state value function can be easily computed from the state-action value function. However, it is intractable in the case of continuous action because Equation (10) needs to evaluate the integral in action space. Therefore, Haarnoja et al. (2018) recommended that the state value function and policy also be separately approximated. Based on the relation (11), the approximation error of the state value function at state ***x*** is given by

$$J_{i}^{V}(\psi_{i},x)=\frac{1}{2}{\left\{V_{i}(x)-E_{u\sim \pi_{i}}\left[Q_{i}(x,\cdot )-\frac{1}{\alpha }\ln\pi (\cdot |x)\right]\right\}}^{2},$$

where the expectation is numerically computed through a Monte Carlo simulation. The loss function for training **ψ**_*i*_ is given by

(14) $$J_{i}^{V}(\psi_{i})=E_{x\sim D}\left[J_{i}^{V}(\psi_{i},\cdot )\right].$$

In the same way, policy parameter **θ**_*i*_ is trained by minimizing the Kullback-Leibler (KL) divergence between the left and right hand sides of Equation (11):

(15) $$J_{i}^{\pi ,\text{RL}}(\phi_{i},x)=D_{\text{KL}}\left(\pi_{i}(\cdot |x)∥\frac{\exp(\alpha Q_{i}(x,\cdot ))}{\exp(\alpha V_{i}(x))}\right),$$

where we need samples drawn from π_*i*_ to evaluate the KL divergence. In addition to the KL divergence, we introduce the behavior cloning loss defined as the KL divergence between the learning and behavior policies:

(16) $$J_{i}^{\pi ,\text{BC}}(\phi_{i},x)=D_{\text{KL}}(\bar{\pi }(\cdot |x)∥\pi_{i}(\cdot |x)).$$

Consequently, the loss function for training **ϕ**_*i*_ is given by

(17) $$J_{i}^{\pi }(\phi_{i})=E_{x\sim D}\left[J_{i}^{\pi ,\text{RL}}(\phi_{i},\cdot )+\eta J_{i}^{\pi ,\text{BC}}(\phi_{i},\cdot )\right],$$

where η is a positive meta-parameter. When η = 0, Equation (17) is identical to the original update rule of Soft Actor-Critic. Note that Information projection (I-projection) is used in Equation (15), and Moment projection (M-projection) is used in Equation (16) (Kober et al., 2013). Although in principle we can select any projection, we believe that Equation (16) is appropriate for the behavior cloning loss because it is averaged over several modes of the policy. In addition, Equation (15) is appropriate because it concentrates on a single mode. π_*i*_ is usually implemented by a Gaussian policy with a single mode, but exp(α*Q*_*i*_(***x***, ·))/exp(α*V*_*i*_(***x***)) may have multiple modes. The update rule of the modified Soft Actor-Critic is given by Algorithm **4**.

**Algorithm 4:** Soft Actor-Critic and Imitation Learning

**Require:** dataset D, inverse temperature η, decay rate τ.
1: Sample a random minibatch of transitions (***x***, ***u***, *r*, ***x***′) from D.
2: Evaluate gradient $\partial J_{i}^{Q}/\partial \theta_{i}$ and update **θ**_*i*_ by stochastic gradient descent.
3: Sample a random minibatch of states ***x*** from D and ***u*** from π_*i*_, respectively.
4: Evaluate gradient $\partial J_{i}^{V}/\partial \psi_{i}$ and update **ψ**_*i*_ by the stochastic gradient descent.
5: Sample a random minibatch of states ***x*** from D and ***u*** from $\bar{\pi }$, respectively.
6: Evaluate gradient $\partial J_{i}^{\pi }/\partial \phi_{i}$ and update **ϕ**_*i*_ by the stochastic gradient descent.
7: Update the parameter of the target network by ${\bar{\psi }}_{i}\leftarrow \tau {\bar{\psi }}_{i}+\left(1-\tau \right)\psi_{i}$.

#### 3.2.3. Deterministic policy gradient and imitation learning

Deterministic Policy Gradient (DPG) (Silver et al., 2014) and its deep version (Lillicrap et al., 2016) are a well-known off-policy reinforcement learning algorithm that can handle continuous actions. Unlike Soft Actor-Critic, DPG does not approximate the state value function. The policy network can also be simplified significantly because it does not need to approximate a continuous probability density function.

The loss function to train *Q*_*i*_ in DPG resembles that in Soft Actor-Critic and is given by Equation (13) whose $J_{i}^{Q}\left(\theta_{i},\text{x},\text{u},r,{\text{x}}^{\prime }\right)$ is replaced with the following equation:

$$J_{i}^{Q}(\theta_{i},x,u,r,x^{\prime })=\frac{1}{2}{\left\{Q_{i}(x,u)-(r+\gamma Q_{i}(x^{\prime },\pi_{i}(x^{\prime });{\bar{\theta }}_{i}))\right\}}^{2},$$

where ${\bar{\theta }}_{i}$ denotes an exponentially moving average of the parameter vector of the target state-action value network and π_*i*_ is a deterministic policy that maps ***x*** to ***u***. DPG evaluates the policy gradient at state ***x*** by

$$\frac{\partial J_{i}^{\pi ,\text{RL}}(\phi_{i},x)}{\partial \phi_{i}}=\frac{\partial Q_{i}(x,u)}{\partial u}{|}_{u=\pi_{i}(x)}\frac{\partial \pi_{i}(x)}{\partial \phi_{i}}.$$

As a result, the policy gradient with behavior cloning loss is computed by

$$\frac{\partial J_{i}^{\pi }}{\partial \phi_{i}}=E_{x\sim D}\left[\frac{\partial J_{i}^{\pi ,\text{RL}}(\phi_{i},\cdot )}{\partial \phi_{i}}-\eta \frac{\partial J_{i}^{\pi ,\text{BC}}(\phi_{i},\cdot )}{\partial \phi_{i}}\right],$$

where $J_{i}^{\pi ,\text{BC}}$ is the same function used by the modified Soft Actor-Critic explained in section 3.2.3. The state value function is simply computed by

$$V_{i}(x)=Q_{i}(x,\pi_{i}(x)).$$

The update rule of the modified DPG is given by Algorithm **5**. One limitation of DPG is that it does not have an explicit exploration mechanism because policy π_*i*_ represents a deterministic function. Therefore, DPG usually introduces a behavior policy that is implemented by an Ornstein-Uhlenbech process (Lillicrap et al., 2016). On the other hand, CRAIL's behavior policy is dynamically constructed by mixing all of the component policies. When DPG is selected as a learning algorithm of CRAIL, at least one learning module with a stochastic policy should be added to promote exploration and discourage premature convergence.

**Algorithm 5:** Deterministic Policy Gradient and Imitation Learning

**Require:** dataset D, inverse temperature η, decay rate τ.
1: Sample a random minibatch of transitions (***x***, ***u***, *r*, ***x***′) from D.
2: Evaluate gradient $\partial J_{i}^{Q}/\partial \theta_{i}$ and update **θ**_*i*_ by the stochastic gradient descent.
3: Sample a random minibatch of states ***x*** from D.
4: Evaluate gradient $\partial J_{i}^{\pi }/\partial \phi_{i}$ and update **ϕ**_*i*_ by the stochastic gradient descent.
5: Update the parameter of the target network by ${\bar{\theta }}_{i}\leftarrow \tau {\bar{\theta }}_{i}+\left(1-\tau \right)\theta_{i}$.

## 4. Experiments

### 4.1. Comparison of CRAIL and CLIS

**Figure 2:**
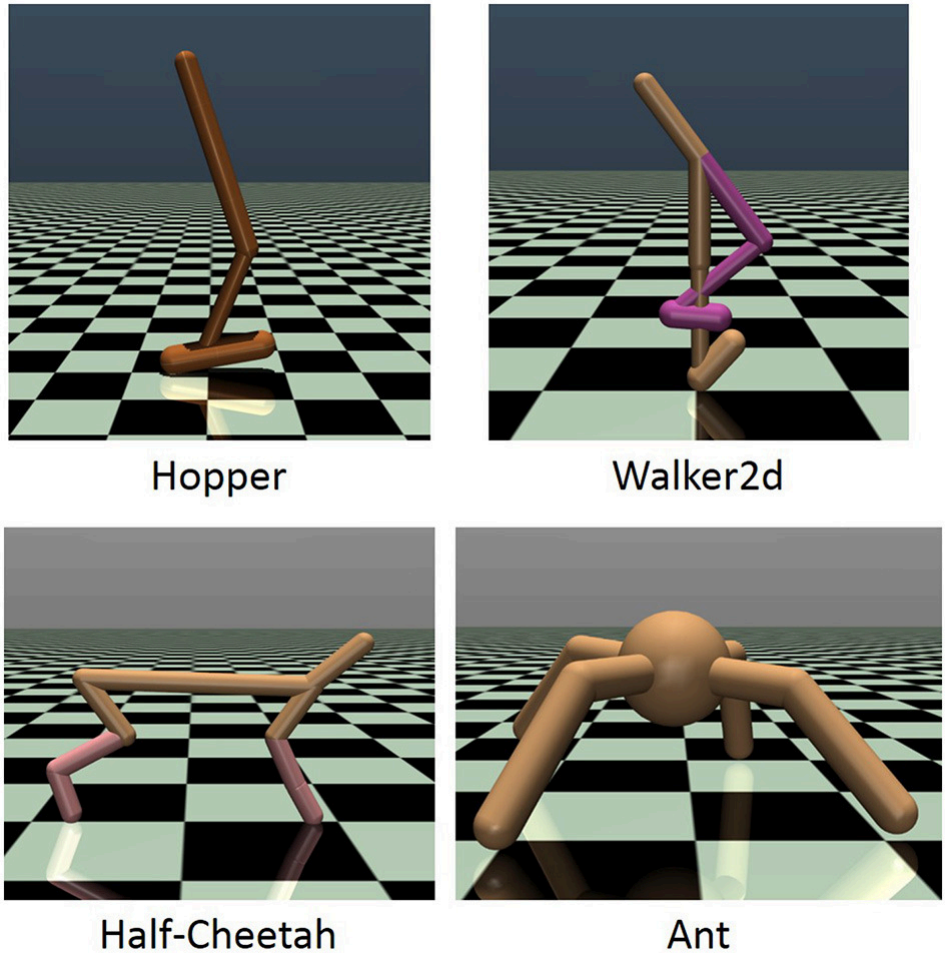
MuJoCo-simulated environments: Hopper-v2, Walker2D-v2, Half-Cheetah-v2, and Ant-v2.

To investigate how CRAIL improves the learning speed, we conducted several computer simulations with four MuJoCo-simulated (Todorov et al., 2012) benchmark tasks: Hopper-v2, Half-Cheetah-v2, Walker2d-v2, and Ant-v2, all of which were provided by the OpenAI gym (Brockman et al., 2016) (Figure 2). Hopper-v2 is a planar monopod, and Walker2d-v2 and HalfCheetah-v2 are planar biped robots. Ant-v2 is a quadruped robot that can move around a three-dimensional environment. The observation and action spaces are shown in Table 1, where the observation vector is used as a state vector. The goal is to move forward as quickly as possible, and the reward function is given by $r\left(\text{x},\text{u}\right)=v_{x}-c||\text{u}|{|}_{2}^{2}$, where *v*_*x*_ is the forward velocity and *c* is a robot-dependent constant. See the supplementary materials of Duan et al. (2016) for the task specifications.

**Table 1:** Environments used in experiments and their state and action spaces.

**Environment**	**Observation space**	**Action space**
Ant-v2	ℝ^111^	[−1.0, 1.0]^8^
HalfCheetah-v2	ℝ^17^	[−1.0, 1.0]^6^
Hopper-v2	ℝ^11^	[−1.0, 1.0]^3^
Walker2d-v2	ℝ^17^	[−1.0, 1.0]^6^

**Figure 3:**
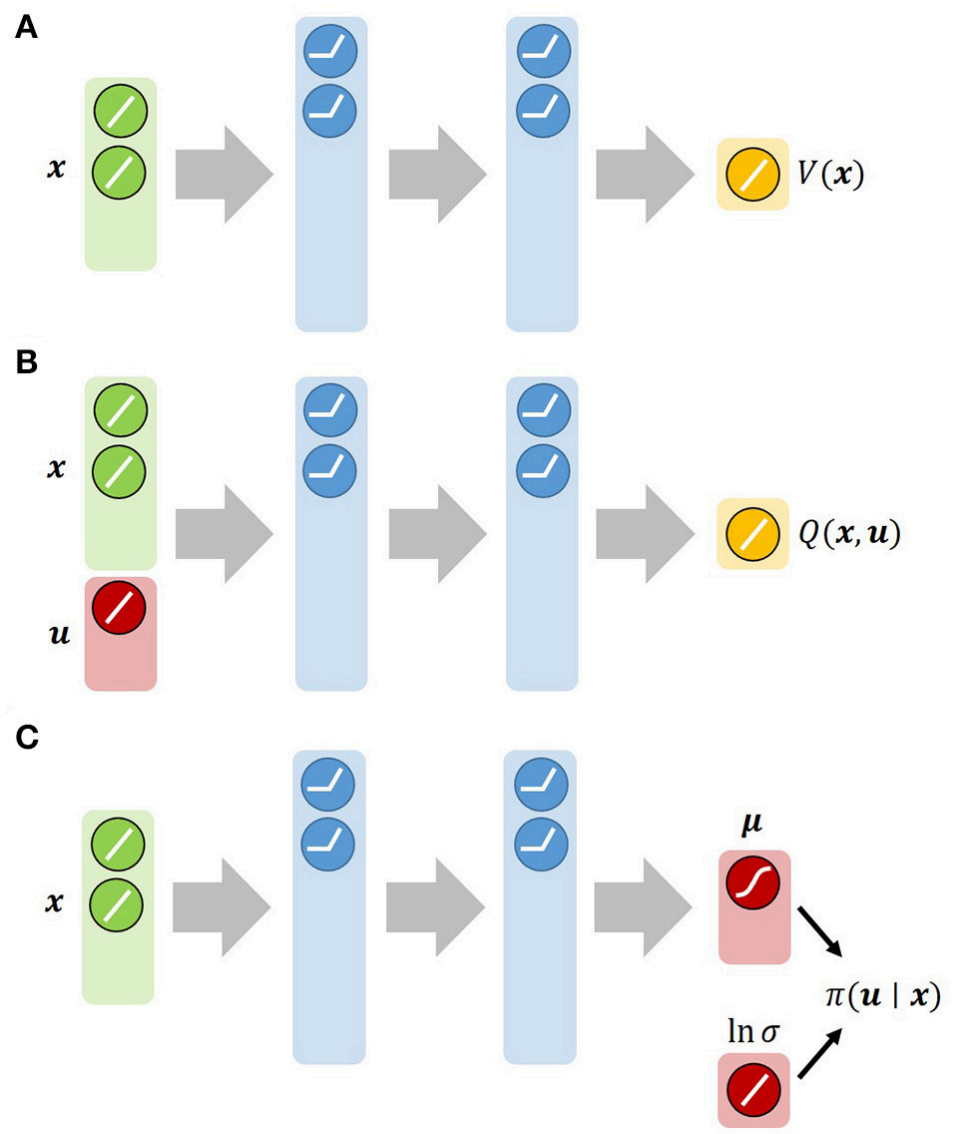
Architectures of neural networks used by Soft Actor-Critic: **(A)** State value function network. **(B)** State-action value function network. **(C)** Gaussian-policy network. We approximate both *V* and *Q* with feed-forward neural networks. π is approximated by a Gaussian policy: $\pi \left(\text{u}|\text{x}\right)=N\left(\text{u}|\mu ,\sigma^{2}\text{I}\right)$, where the mean **μ** is given by a neural network and the log-standard deviation ln σ is parameterized by a global vector independent of the state.

We prepared two function approximators, Neural Network (NN) and normalized Radial Basis Function (RBF), and Table 2 shows their network architectures. For example, the module using the RBF networks represents *V*_*i*_ by 64 normalized radial basis functions by

$$V_{i}(x;\psi_{i})=\sum_{j=1}^{N_{i}}\psi_{i,j}b_{i,j}(x),$$

where *N*_*i*_ and ψ_*i, j*_ respectively denote the number of basis functions and the *j*-th element of **ψ**_*i*_ and *b*_*i, j*_(***x***) is the basis function defined by

$$b_{i,j}(x)=\frac{a_{i,j}(x)}{\sum_{j^{\prime }=1}^{N_{i}}a_{i,j^{\prime }}(x)},\;a_{i,j}=\exp\left(-\|s_{i,j}^{⊤}(x-c_{i,j}){\|}_{2}^{2}\right),$$

where *a*_*i, j*_ is a Gaussian activation function with parameters **s**_*i, j*_ and **c**_*i, j*_. Since **s**_*i, j*_ and **c**_*i, j*_ were determined by a heuristic rule (Morimoto and Doya, 2001), *V*_*i*_ is interpreted as a linear neural network. Therefore, the module with the RBF networks is expected to learn faster than that with the nonlinear neural networks. Figure 3 represents the architectures that approximate π_*i*_, *V*_*i*_ and *Q*_*i*_ needed by the Soft Actor-Critic. Each was implemented by a feed-forward neural network with a Rectified Linear Unit (ReLU) as a nonlinear activation function of the hidden layers. In the first experiment, we chose three learning algorithms, Soft Actor-Critic, Deterministic Policy Gradient, and REINFORCE with importance sampling. We prepared 2 × 3 = 6 modules as a result. To apply Algorithm **3** to this non-episodic task, the horizon length *T* is set to 300.

**Table 2:** Network architectures of approximator in the first and the second experiments: For example, RBF module approximates *Q*_*i*_ by 64 basis functions, and NN module approximates two-layer feed-forward neural network consisting of (400, 300) hidden units.

**Approximator**	**V**	**Q**	**π**
RBF	(64)	(64)	(64)
NN	(64, 64)	(400, 300)	(400, 300)

CRAIL was given the above six modules for parallel training. We also tested the six modules separately in addition to CLIS as baseline performances, where CLIS also used multiple importance sampling instead of an independent type because the original CLIS worked very poorly due to the unboundedness of the importance-sampling weight ratio. Note that the original CLIS selects one learning module at the beginning of each episode, and utilizes a truncated importance sampling ratio given by

$${\hat{\rho }}_{i}(h)=\min\left(\prod_{t=1}^{T}\frac{\pi_{i}(u_{t}|x_{t})}{\pi_{\text{selected}}(u_{t}|x_{t})},C\right),$$

where π_selected_ is the policy of the selected module and *C* is a positive constant determined by the experimenters. Although ${\hat{\rho }}_{i}\left(h\right)$ is upper-bounded, it is not trivial to tune *C* in practice. In addition, CLIS does not consider behavior cloning loss. Therefore, CLIS evaluated in the experiments uses Equation (4) as the importance weight. In this case, CLIS is identical to CRAIL with η = 0. Each method was evaluated in ten simulation runs, each of which was comprised of 2,000 episodes.

**Figure 4:**
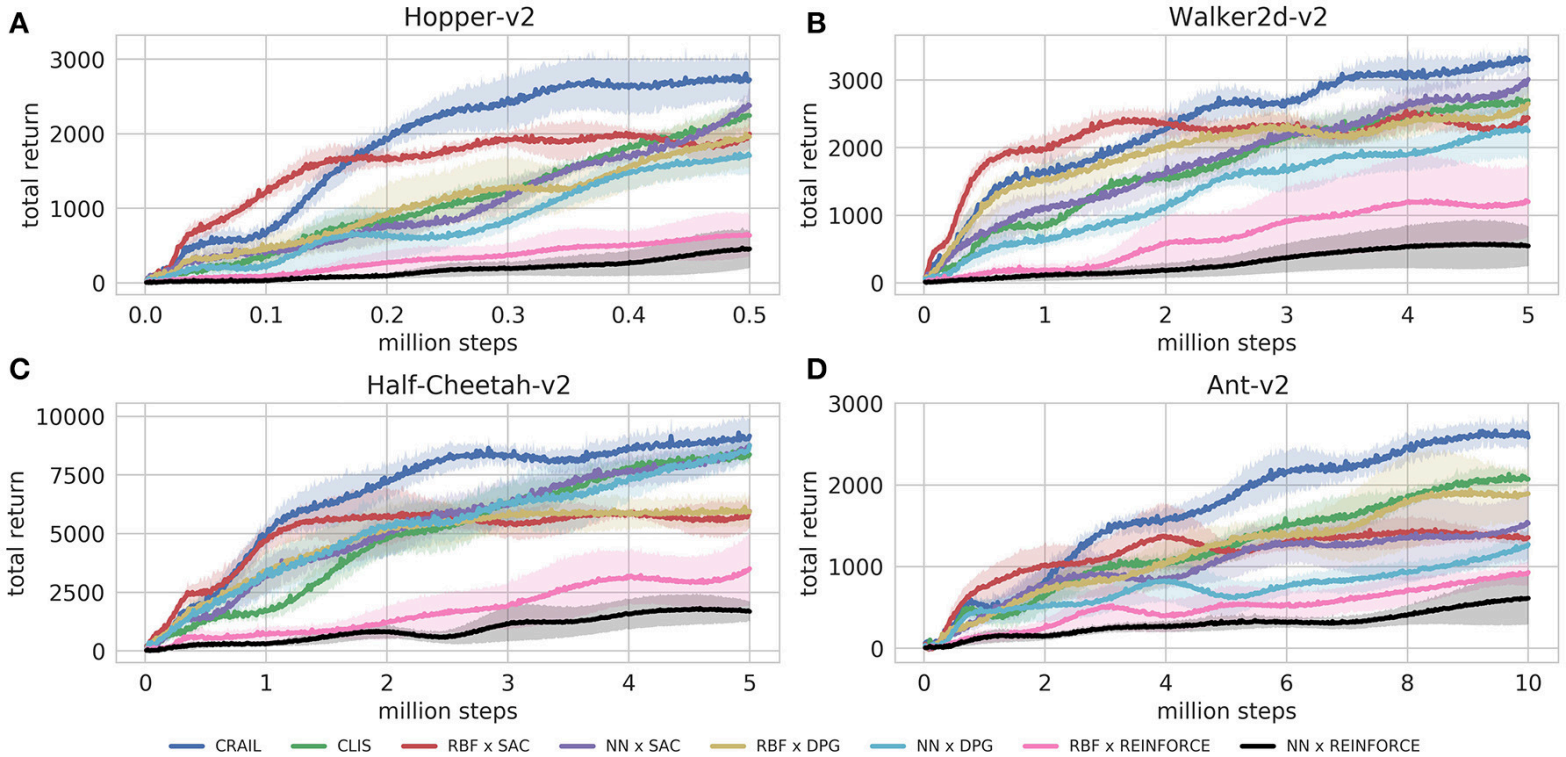
Training curves on continuous control benchmarks: Performance was evaluated by cumulative rewards in 10 episodes for each learning module.

**Figure 5:**
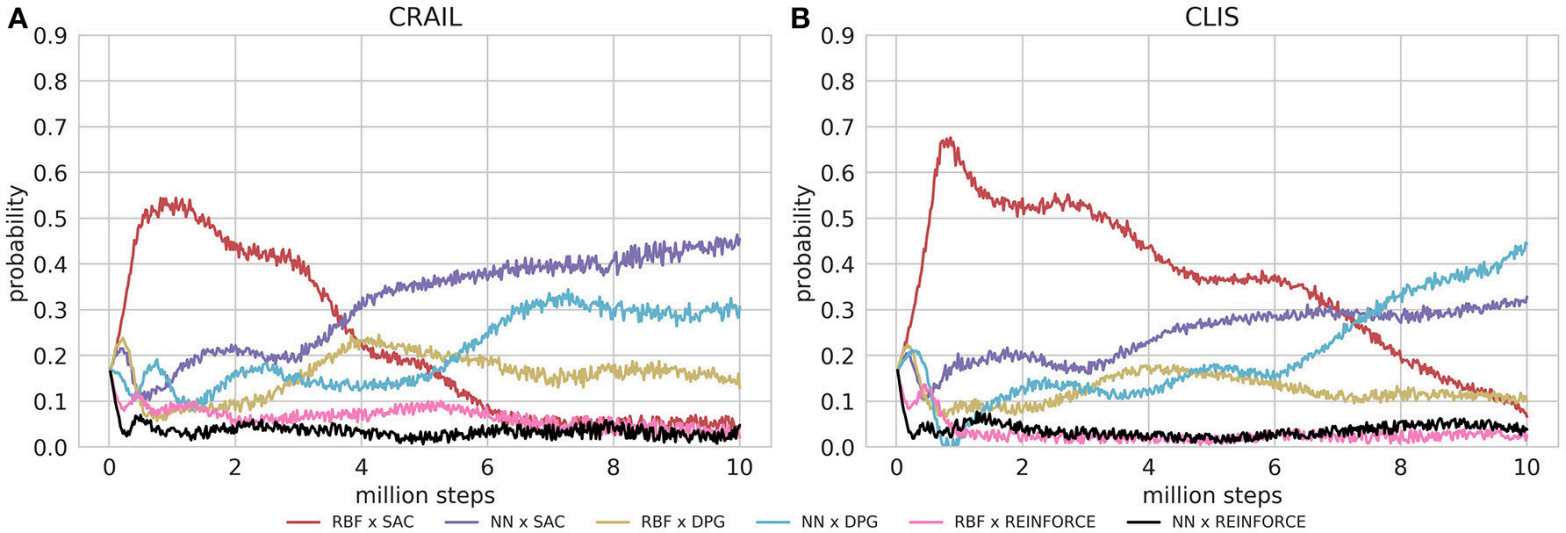
Probabilities for changing learning modules during learning process: **(A)** Results obtained by CLIS architecture. **(B)** Results obtained by CL without importance sampling.

Figure 4 shows the learning performance of CRAIL, CLIS, and the six component modules, and we found that CRAIL learned faster than CLIS and the six modules trained separately on all the benchmark tasks. On the other hand, the learning performance of CLIS resembled that of the NN × SAC module. The RBF × SAC module showed the best learning curves on all the tasks at the early stage of learning, but its performance saturated before reaching a sufficient level because the normalized RBF networks could not precisely approximate the value functions and the policy as well as the neural networks. On the contrary, the NN policies trained by SAC or DPG learned very slowly, and their performance was much worse than RBF × SAC at the early stage of learning. The modules trained by REINFORCE needs a set of sequences, and therefore, they learned slower than the actor-critic methods such as DPG and Soft AC. As a result, the REINFORCE modules achieved worse performance, and the probabilities remained low during learning. Figures 5A,B respectively show the mixing weights ${\left\{\alpha_{i}\right\}}_{i=1}^{6}$ during the learning of Ant-v2 computed by CRAIL and CLIS. The probability of selecting the RBF × SAC module increased rapidly at the early stage of learning in both cases. However, CRAIL tended to gradually select the NN × SAC module after about four million steps, and CLIS continued to choose the RBF × SAC module's policy most frequently until about six million steps.

### 4.2. Adaptation to changes in the environment

Next, we experimentally tested the capability of adaptation to changes in the environment by changing the mass of the body of HalfCheetah-v2 from 6.36 (original) to 6.36 × 3 [kg] at the 5 millionth step. In this experiment, both CRAIL and CLIS possessed the same six learning modules used in the previous experiment. Each method was evaluated in ten simulation runs, each of which was comprised of 2,000 episodes.

**Figure 6:**
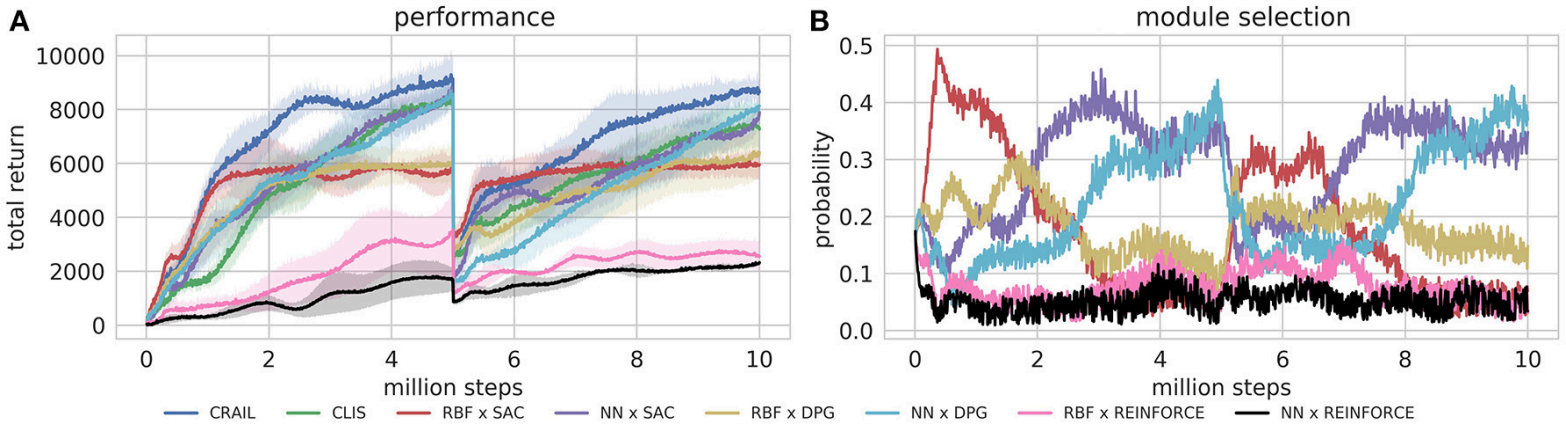
Training curves on episodic Half-Cheetah-v2 task, in which body's mass was changed at 5 millionth step.

Figures 6A,B respectively show the cumulative rewards and module selection probability in each step. Note that the first half of Figure 6A is identical to Figure 4C. When the mass was changed at 5 millionth steps, the performance of the CRAIL, CLIS, and NN policies decreased significantly. However, the RBF policies maintained the pole without considerable deterioration in performance compared with the NN policies because the number of weights was smaller. In other words, the performances of the NN policies deteriorated drastically because their policies were fine-tuned for a particular weight. Therefore, the probability of selecting RBF × SAC increased temporarily from about 5 to 6.5 million steps. CRAIL prevented the body from falling and trained NN × SAC and NN × DPG by appropriately selecting RBF × SAC, as shown in Figure 6B.

### 4.3. Introducing a fixed policy

To investigate how CRAIL exploits a deterministic stationary policy, we added a CPG-based policy as prior knowledge to control HalfCheetah-v2 because periodic motion is quite useful to generate walking behaviors and many previous studies exist (Ijspeert, 2008) in this field. Since CRAIL uses multiple importance sampling, it is straightforward to use the deterministic policy as one of the sampling policies. Note that the CPG-based policy has internal states because the oscillator is implemented by a differential equation. Therefore, we selected the REINFORCE algorithm with importance sampling described in section 3.2.1 and Algorithm **2** in this experiment because the evaluation of deterministic policies with internal states is difficult in stepwise update rules.

As learning modules, we prepared three network architectures that are commonly seen in the literature (Henderson et al., 2018) as shown in Table 3 to implement a stochastic policy. We used a ReLU nonlinear activation function. Note that the REINFORCE algorithm does not need *Q*_*i*_. In addition, a deterministic stationary policy based on central pattern generators was prepared as prior knowledge, which was implemented by the modified Hopf oscillator (Uchibe and Doya, 2014). Since CRAIL uses multiple importance sampling, it is straightforward to use the deterministic policy as one of the sampling policies.

**Table 3:** Network architectures of approximator in the third experiments: We denote the hidden layer sizes of a two-layer feedforward neural network as *(N, M)*.

**Approximator**	**V**	**π**
BASE	(64, 64)	(64, 64)
WIDE	(64, 64)	(400, 300)
DEEP	(64, 64)	(100, 50, 25)

*For example, the WIDE module approximates *V*_i_ and π_i_ by (64, 64) and (400, 300), respectively*.

**Figure 7:**
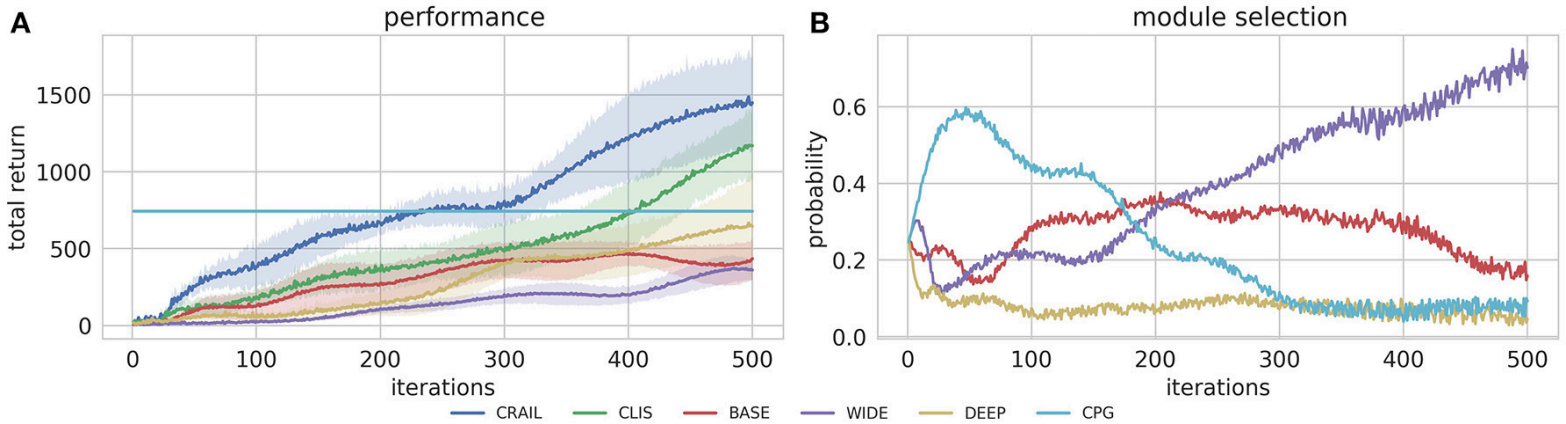
Training curves on Half-Cheetah-v2 task, in which fixed stationary policy was used as prior knowledge.

In addition to evaluate the CRAIL's performance, we tested the four modules separately. Figure 7A shows that CRAIL learned much faster than the component modules trained alone. Since REINFORCE learns very slowly due to its simplicity (Duan et al., 2016), 500 iterations were insufficient to overcome the CPG-based controller. Figure 7B shows the mixing weights during the learning computed by CRAIL. The probability of selecting the CPG-controller module increased rapidly at the early stage of learning. Then, CRAIL tended to select the BASE module and the probability of selecting it was the highest among the NN modules from about 90 to 170 iterations. Finally, the WIDE module was frequently selected at the later stage of learning. The DEEP module trained alone achieved the highest performance among the three neural network policies. However, the probability of selecting it remained low during learning. Note that the original CLIS cannot utilize the deterministic policy because the importance weight ratio becomes infinity.

## 5. Discussion

This paper proposed modular reinforcement learning (CRAIL), which collects task-relevant samples using multiple heterogeneous policies. One interesting feature of CRAIL is that a complex RL system can learn faster with the help of a simple RL system that cannot achieve the best performance. Experimental results also suggested that CRAIL efficiently adapted to changes in the learning conditions because it automatically selected simple modules with fewer parameters.

CRAIL implicitly assumes that state value functions are not initialized optimistically. Suppose that the reward function is always non-positive, and the state value functions are initialized to zero. In this case, some modules that are not selected by Equation (1) may have *V* values that are consistently higher than the selected modules. In this case, CRAIL selects the worst module if the inverse temperature is not tuned appropriately. As one possible extension to overcome this difficulty, the mixing weights are also trained by reinforcement learning in which the value functions are used as priors.

In the current implementation, since all the learning modules are prepared in advance CRAIL cannot obtain good performance if all of them are inappropriate for the given task. To design appropriate learning modules, we need to develop a mechanism to add or delete learning modules based on the selection probabilities calculated by Equation (1). If a simple learning module has a low probability for a long time, it can be replaced by a complicated module. This allows CRAIL to flexibly test heterogeneous modules without increasing computational costs. To overcome this problem, we consider an asynchronous version of the algorithms.

We did not address the effects of computational costs on the learning modules. Updating the parameters of the RBF networks was accomplished considerably faster than for the deep neural networks, but the modules with the RBF networks had to wait until the modules with deep neural networks completed their computations. In general, the sampling rate significantly affects the original performance of a robot. For example, the robot should reduce its moving speed when it uses a complex module. However, the effects of the differences in sampling rates have not been scrutinized.

One interesting future topic is the use of multiple meta-parameters. CRAIL has some meta-parameters used in an RL system, and their settings, such as the learning rate, the inverse temperature that controls the randomness in action selection, and the discount factor for future reward prediction, are crucial to perform a task successfully. A possible scenario is that when a small discount factor can be used in the initial learning process, a module with a larger discount factor can be selected as the learning progresses. We have not yet identified the tasks and situations in which different discount factors play an important role for accelerating the learning speed, but in the future we will seek good examples for this topic.

## Author contributions

EU conceived, designed the research, performed the experiment, analyzed its results, and wrote the paper.

### Conflict of interest statement

The author declares that the research was conducted in the absence of any commercial or financial relationships that could be construed as a potential conflict of interest.
